# DAPK2 regulates oxidative stress in cancer cells by preserving mitochondrial function

**DOI:** 10.1038/cddis.2015.31

**Published:** 2015-03-05

**Authors:** C R Schlegel, M L Georgiou, M B Misterek, S Stöcker, E R Chater, C E Munro, O E Pardo, M J Seckl, A P Costa-Pereira

**Affiliations:** 1 Department of Surgery and Cancer, Imperial College London, Faculty of Medicine, Hammersmith Hospital Campus, ICTEM, Du Cane Road, London W12 0NN, UK

## Abstract

Death-associated protein kinase (DAPK) 2 is a serine/threonine kinase that belongs to the DAPK family. Although it shows significant structural differences from DAPK1, the founding member of this protein family, DAPK2 is also thought to be a putative tumour suppressor. Like DAPK1, it has been implicated in programmed cell death, the regulation of autophagy and diverse developmental processes. In contrast to DAPK1, however, few mechanistic studies have been carried out on DAPK2 and the majority of these have made use of tagged DAPK2, which almost invariably leads to overexpression of the protein. As a consequence, physiological roles of this kinase are still poorly understood. Using two genetically distinct cancer cell lines as models, we have identified a new role for DAPK2 in the regulation of mitochondrial integrity. RNA interference-mediated depletion of DAPK2 leads to fundamental metabolic changes, including significantly decreased rate of oxidative phosphorylation in combination with overall destabilised mitochondrial membrane potential. This phenotype is further corroborated by an increase in the production of mitochondrial superoxide anions and increased oxidative stress. This then leads to the activation of classical stress-activated kinases such as ERK, JNK and p38, which is observed on DAPK2 genetic ablation. Interestingly, the generation of oxidative stress is further enhanced on overexpression of a kinase-dead DAPK2 mutant indicating that it is the kinase domain of DAPK2 that is important to maintain mitochondrial integrity and, by inference, for cellular metabolism.

Death-associated protein kinase (DAPK) 2 shares a high level of homology within its kinase domain with the other two DAPK family members, DAPK1 (DAPk) and DAPK3 (ZIPK/DLK). Since the identification of DAPK1 by Kimchi and co-workers^1^ numerous studies have shown that DAPK1 functions as a tumour suppressor, is linked to key events in autophagy and is involved in mitochondrial maintenance^2^ and metabolism.^3^ DAPK2, which was characterised in 1999,^4^ is significantly smaller than DAPK1, and it lacks ankyrin repeats, the cytoskeletal binding domain and the death domain, all of which are part of DAPK1's unique structure.^1^ Several functions have been ascribed to DAPK2 and they often coincide with those of DAPK1. Like DAPK1, DAPK2 is also involved in the formation of autophagic vesicles,^5, 6^ modulation of receptor induced cell death^7, 8, 9^ and several modes of intrinsic apoptotic cell death.^6^ While epigenetic silencing of DAPK1 has been reported in many different human cancers,^10, 11^ DAPK2 appears to be silenced mainly in haematological disorders,^12^ although it has been shown to modulate TRAIL-induced apoptosis in several cancer cell lines of non-haematological origin.^9^ Most approaches used for studying the role of DAPK2 used tagged DAPK2 and it is, therefore, still unclear whether these functions are also carried out by the native protein, expressed at much lower, endogenous, levels.

DAPK1 has been shown to regulate mitochondrial integrity and to modulate the mitochondrial membrane potential^2^ but, to the best of our knowledge, no work has been carried out in this respect with regard to DAPK2. Since DAPK1 and DAPK2 appear to share many functions and both are thought to reside, at least partially, in the mitochondria, we hypothesised that DAPK2 depletion regulated mitochondrial metabolism. Mitochondrial dysfunction is characterised by the induction of reactive oxygen species (ROS) in the cell.^13^ Ultimately, dysfunctional mitochondria can no longer be powerhouses of use to the cell and are, therefore, targeted for degradation. Alternatively, their membranes can depolarise leading to the release of cytochrome *c*, an early apoptotic process.^14^ Using two distinct cancer cell types, namely U2OS osteosarcoma and A549 non-small cell lung cancer cells,^9, 15^ we show that DAPK2 depletion increases the levels of intracellular ROS, leads to mitochondrial depolarisation and impairs mitochondrial metabolism. DAPK2 thus exerts metabolic and mitochondria-regulating functions, which have not been described to date and that can explain why it is downregulated in haematological malignancies,^12, 16, 17^ and involved in modulating death-inducing signalling in solid tumours.^9^

## Results

### RNAi-mediated ablation of DAPK2 induces oxidative stress and activates mitogen-activated protein kinases

**Figure 1:**
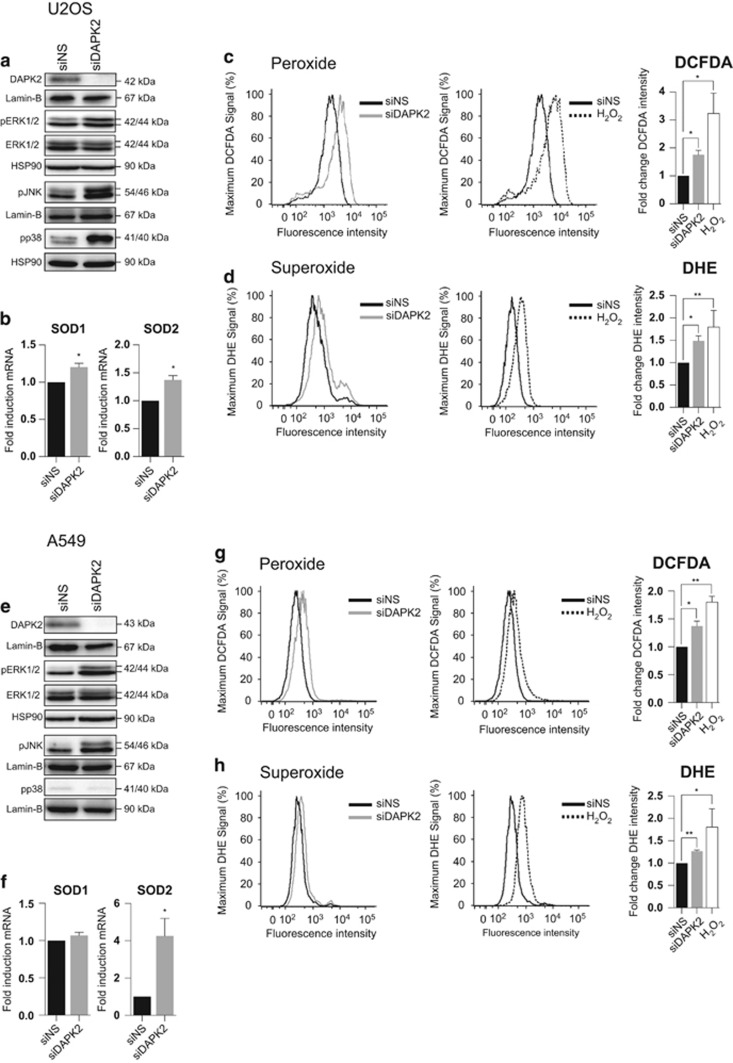
DAPK2 depletion induces oxidative stress, phosphorylation of MAPKs and transcription of SODs. U2OS (**a**–**d**) and A549 (**e**–**h**) cells were transfected with either siNS or DAPK2 siRNA. Forty-eight hours after transfection, the efficacy of DAPK2 silencing and phosphorylation of ERK1/2, JNK and p38 were assessed by SDS-PAGE/qWB using Lamin B and HSP90 as loading controls (**a** and **e**), and the induction of SOD1 and SOD2 mRNA was measured by qPCR (**b** and **f**). Data represent mean±S.E.M. of three independent experiments. Statistical analyses were done using Student's *t*-test (paired, one tailed) (*P*<0.05). Oxidative stress was detected by flow cytometry. Cells were transfected with siNS or siDAPK2 for 48 h, and H_2_O_2_ treatment (0.5 mM, 24 h) was used as a positive control for ROS production. The DCFDA probe was used to detect general ROS in U2OS (**c**) and A549 cells (**g**), whereas O_2_^•−^ anions were detected in U2OS (**d**) and A549 cells (**h**) using the DHE probe. Staining intensity was quantified using geometric means of three independent experiments and plotted as fold change in relation to the siNS-transfected cells. Statistical analysis was done using Student's *t*-test (paired, one tailed) (*P<0.05, **P<0.01)

Although DAPK2 is structurally significantly different from DAPK1, their kinase domains are highly homologous.^18^ As DAPK1 has been shown to be involved in mitochondrial regulation,^2^ we asked whether DAPK2 could also modulate the level of cellular oxidative stress. For that purpose, DAPK2 was targeted with a pool of short interfering (si) RNA oligonucleotides (henceforth referred to as siDAPK2), previously validated in our laboratory (Supplementary Figure S1 and Schlegel *et al*^9^). U2OS osteosarcoma and A549 lung cancer cells were transfected with either a control non-targeting siRNA oligonucleotide (siNS) or with siDAPK2 and proteins were analysed by sodium dodecyl sulphate-PAGE (SDS-PAGE). Quantitative western blots (qWB) in Figure 1 show the efficiency of the knockdown of DAPK2 in U2OS (Figure 1a) and A549 (Figure 1e) cells, when compared with the control siNS-transfected cells.

Oxidative stress was studied by flow cytometry in cells transfected with siNS, siDAPK2 or challenged with H_2_O_2_ (positive control for the production of ROS). General oxidative stress was analysed using the chloromethyl 2′,7′-dichlorodihydrofluorescein diacetate (CM-H_2_DCFDA, henceforth referred to as DCFDA) probe (Figures 1c and g) and superoxide production was assessed using the dihydroethidium (DHE) probe (Figures 1d and h). The fluorescence of cells transfected with siDAPK2 or treated with H_2_O_2_ was compared to that of control cells (siNS). The histograms depict typical cell profiles obtained in each experiment and the bar charts on the right conglomerate mean percentage of fluorescence normalised to control cells±S.E.M. from three independent experiments. Both DAPK2 depletion and H_2_O_2_ treatment resulted in an increase in general oxidative stress in U2OS (Figure 1c) and A549 (Figure 1g) cells. The same was observed with regard to the generation of superoxides, which was elevated on siDAPK2 depletion and on treatment with H_2_O_2_ in both U2OS (Figure 1d) and A549 cells (Figure 1h).

Having observed a clear induction of ROS (H_2_O_2_ and O_2_^•−^) on silencing DAPK2, we asked whether this led to the activation of mitogen-activated protein kinases (MAPKs), which are known to be activated by oxidative stress as part of a pro-survival response.^19^ Hence, WB membranes (Figures 1a and e) were probed with antibodies raised against the phosphorylated forms of ERK1/2, p38 and JNK. Depletion of DAPK2, when compared with the siNS control, led to clear phosphorylation/activation of ERK1/2^Thr202/Tyr204^ and JNK^Thr183/Tyr185^ in U2OS and A549 cells, whereas phosphorylation of p38^Thr180/Tyr182^ was only observed in U2OS cells but not in A549 lung cancer cells (Figure 1a *versus* Figure 1e).

Superoxide anion (O_2_^•−^) is a potentially damaging free radical, which is converted into less reactive hydrogen peroxide (H_2_O_2_) by enzymes of the superoxide dismutase (SOD) family. To analyse the effect of DAPK2 depletion on the expression of SOD1, the predominant cytoplasmic SOD, and mitochondrial SOD2, cells were transfected as before and RNA was subsequently extracted. There was significant, *albeit* small (<1.5-fold), upregulation of both SOD1 and SOD2 mRNA in U2OS cells (Figure 1b). In contrast, in A549 cells, SOD1 mRNA was not induced but SOD2 mRNA was greatly increased (greater than fourfold; Figure 1f).

### DAPK2 knockdown increases the levels of mitochondrial O_2_^•−^ and leads to spontaneous mitochondrial membrane depolarisation

**Figure 2:**
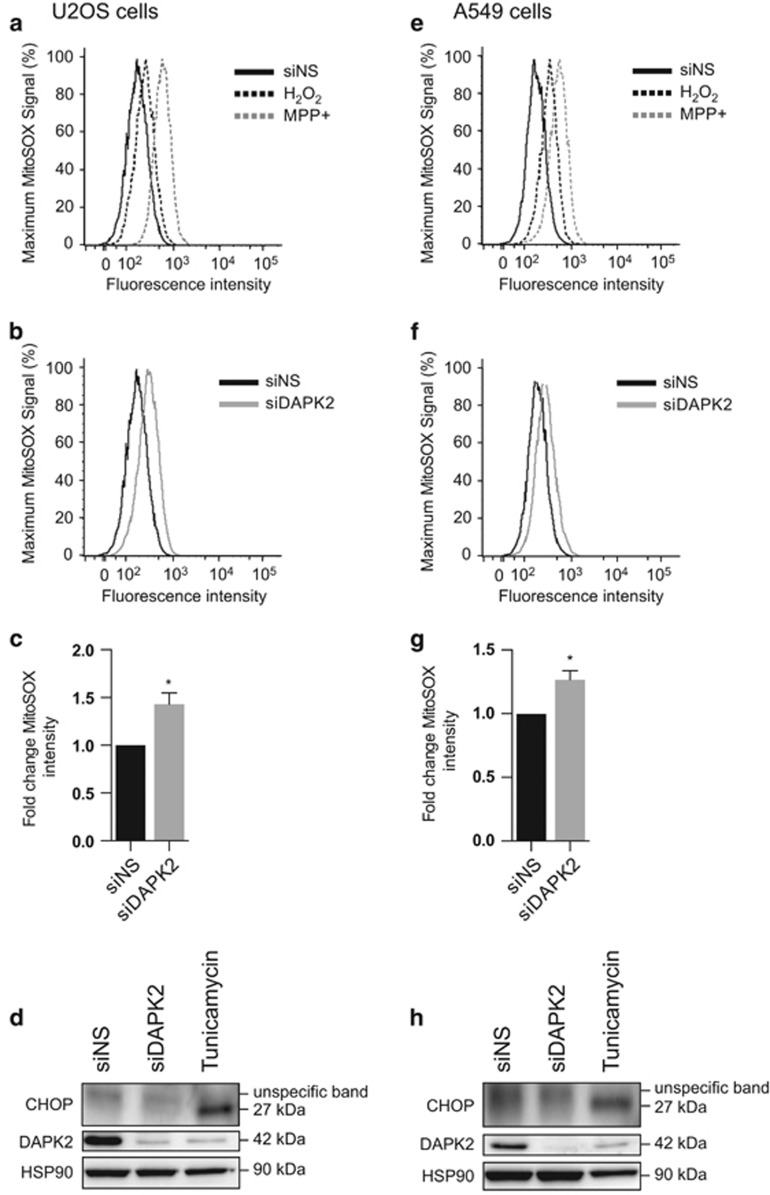
DAPK2 knockdown triggers mitochondrial O_2_^•−^ production. U2OS (**a**–**d**) and A549 (**d**–**h**) cells were transfected with either siNS or DAPK2 siRNA. Forty-eight hours later, mitochondrial O_2_^•−^ levels were assessed using the MitoSOX Red probe. U2OS (**a** and **b**) and A549 (**e** and **f**) cells were also treated with H_2_O_2_ (0.5 mM) or MPP^+^ (1 mM), which were used as positive controls, for 24 h. The average of geometric means of four independent experiments was plotted as fold change (siNS *versus* siDAPK2) (**c** and **g**). Statistical analysis was done using Student's *t*-test (paired, two tailed) (*P<0.05). ER stress was assessed 48 h after siRNA transfection by SDS-PAGE/qWB using CHOP expression as a read-out (**d** and **h**). The induction of CHOP protein following treatment with tunicamycin (0.5 *μ*g/ml) served as positive control and HSP90 expression was used as a loading control

Silencing DAPK2 in two different cell lines led to upregulation of cellular ROS, downstream activation of MAPKs and upregulation of mitochondrial SOD2, whereas SOD1 was only slightly upregulated in one of the cell lines (U2OS). We, therefore, asked whether the source of oxidative stress were mitochondria. Indeed, the production of ATP by oxidative phosphorylation is a major source for mitochondrial ROS, and mitochondrial proton and electron leaks can impact on mitochondrial coupling efficiency and lead to increased production of mitochondrial ROS.^20^ MitoSOX Red (Molecular Probes) was used to assess mitochondrial O_2_^•−^ levels since it selectively targets mitochondria and is exclusively oxidised by O_2_^•−^ (Figure 2). Cells were transfected as before and treatments with H_2_O_2_ or the mitochondrial complex I inhibitor 1-methyl-4-phenylpyridinium (MPP^+^) iodide served as positive controls for the experiment. Unsurprisingly, both treatments increased the levels of mitochondrial O_2_^•−^ (Figures 2a and e). As hypothesised, RNA interference (RNAi)-mediated depletion of DAPK2 resulted in a small but statistically significant increase of mitochondrial O_2_^•−^ in U2OS (Figures 2b and c) and A549 cells (Figures 2f and g) compared with siNS-transfected cells.

Another potential source for cellular ROS is the endoplasmic reticulum (ER) where the transcription factor CCAAT-enhancer-binding protein homologous protein (CHOP) is specifically activated on ER stress.^21^ To induce ER stress we used tunicamycin,^22^ which induced the expression of CHOP in both U2OS (Figure 2d) and A549 cells (Figure 2h). In contrast, DAPK2 knockdown did not increase CHOP expression in either cell line (Figures 2d and h).

**Figure 3:**
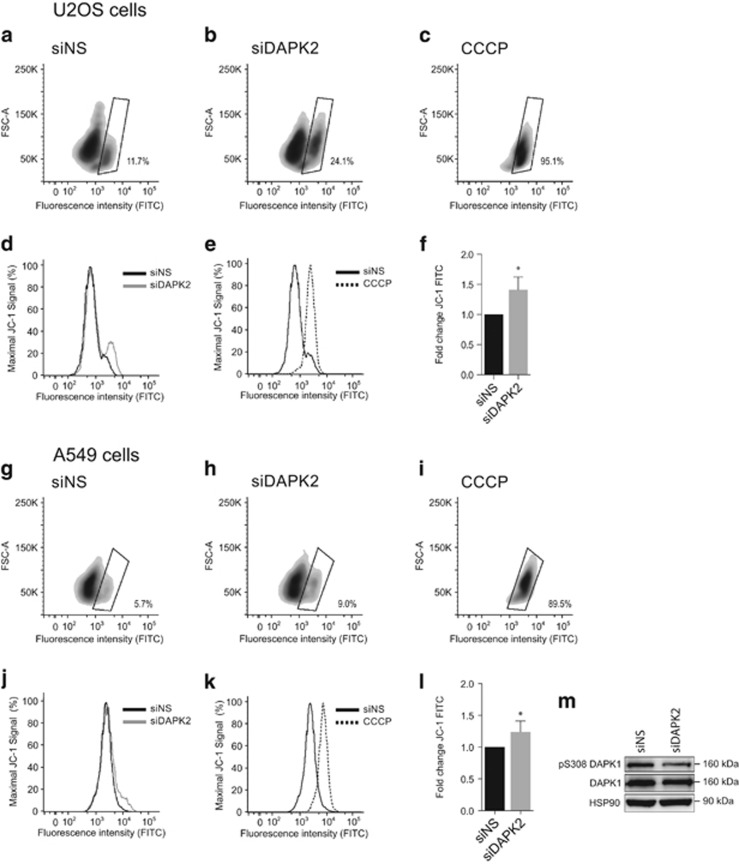
Genetic ablation of DAPK2 increases the rate of spontaneous mitochondrial membrane depolarisation using the JC-1 probe. U2OS (**a**–**f**) and A549 (**g**–**m**) cells were transfected with either siNS or siDAPK2. Forty-eight hours after transfection cells were incubated with JC-1 and the fluorescence of J-aggregates or monomers was measured in red and green fluorescence channels by flow cytometry. CCCP (50 *μ*M) was used to induce complete mitochondrial depolarisation (**c** and **i**) and to set appropriate gates in U2OS (**a**–**c**) and A549 cells (**g**–**i**) used for the quantification of mitochondrial depolarisation following transfection with siNS (**a** and **g**), or siDAPK2 (**b** and **h**). Overall green fluorescence (FITC) data is also presented in histograms (U2OS: **d** and **e**; A549: **j** and **k**). Staining intensity was quantified using geometric means of three independent experiments and plotted as fold change (**f** and **l**). Data represent mean±S.E.M. of three independent experiments and the statistical analysis was done using Student's *t*-test (paired, one tailed) (*P<0.05). The phosphorylation of DAPK1 on Ser308 and DAPK1 expression levels were analysed in A549 lung cancer cells by SDS-PAGE/qWB, using HSP90 as a loading control (**m**)

**Figure 4:**
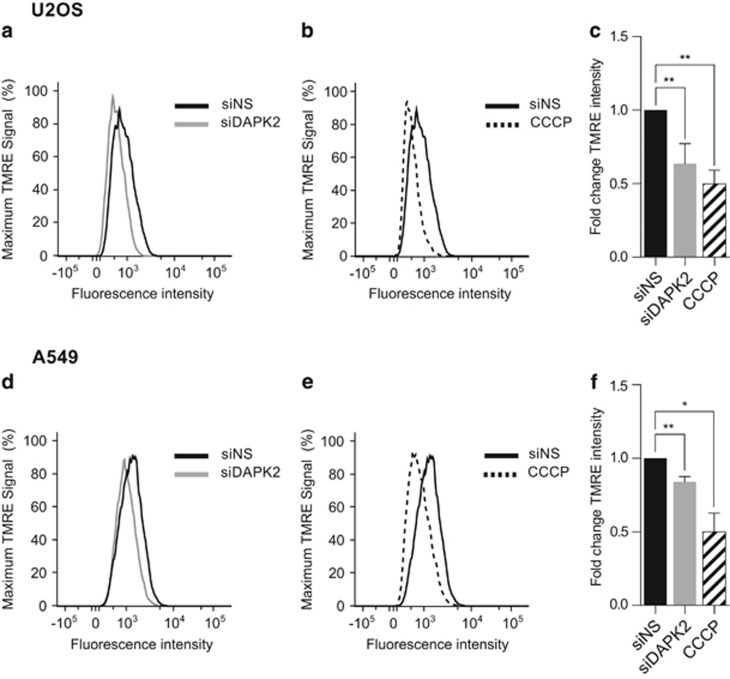
Measurement of spontaneous mitochondrial membrane depolarisation after transfection with siDAPK2 using the TMRE probe. U2OS (**a**–**c**) and A549 (**d**–**f**) cells were transfected with either siNS or siDAPK2. Forty-eight hours after transfection cells were incubated with TMRE and the fluorescence intensity in the red fluorescence channel (PE) was measured by flow cytometry. CCCP (50 *μ*M) was used to induce mitochondrial depolarisation (**b** and **e**). Staining intensity was quantified using geometric means of three independent experiments and plotted as fold change (**c** and **f**). Data represent mean±S.E.M. of three independent experiments and the statistical analysis was done using Student's *t*-test (paired, one tailed) (*P<0.05, **P<0.01)

Elevated levels of mitochondrial O_2_^•−^ can be both a cause and a consequence of mitochondrial depolarisation,^23^ which is involved in apoptosis and inflammation.^24, 25^ To study the effect of DAPK2 depletion on mitochondrial membrane potential (Δ*ψ*m), the JC-1 probe was used. U2OS and A549 cells were transfected with either siNS, or siDAPK2 and, as a control for Δ*ψ*m depolarisation, cells were treated with carbonyl cyanide 3-chlorophenylhydrazone (CCCP), and then analysed by flow cytometry. Two distinct cell populations were identified after gating the depolarised population that resulted from CCCP treatment: one with intact mitochondria and another that harboured cells with depolarised mitochondria. Depletion of DAPK2 led to ~50% more depolarised mitochondria in both U2OS (Figures 3a–c) and A549 cells (Figures 3g–i), when compared with control cells. This pattern can also be seen in the histograms in Figures 3d, e, j and k. After normalising the absolute fluorescence of cells on DAPK2 knockdown to that of siNS-transfected cells, a significant overall increase in green fluorescence, a read-out for decreased Δ*ψ*m, was measured in both cell lines (Figures 3f and l), suggesting that DAPK2 ablation increased spontaneous mitochondria depolarisation. This was confirmed using tetramethylrhodamine ethyl ester (TMRE), another mitochondrial probe (Figure 4).

Treatment with mitochondrial complex inhibitors leads to the activation of DAPK1 and a decrease in Δ*ψ*m^2^. As shown in Figure 3m A549 cells depleted of DAPK2 have reduced DAPK1 phosphorylated on S308, which is an inactivating phosphorylation event that modulates DAPK1 activity. This suggested that silencing DAPK2 led to the activation of DAPK1. Interestingly, U2OS cells did not express DAPK1 protein or mRNA (data not shown). Thus, although mitochondrial depolarisation in A549 cells might be associated with activation of DAPK1, in U2OS cells this is unlikely.

### Altered mitochondrial integrity leads to metabolic changes

The increase in mitochondrial O_2_^•−^ production and spontaneous mitochondrial depolarisation after DAPK2 silencing suggested that mitochondria were the likely source of ROS. The data were consistent with a significant impairment of mitochondrial integrity in response to DAPK2 depletion and, in the case of A549 lung cancer cells, activation of DAPK1. The metabolic consequences of these mitochondrial alterations in DAPK2-depleted cells were, therefore, investigated next.

**Figure 5:**
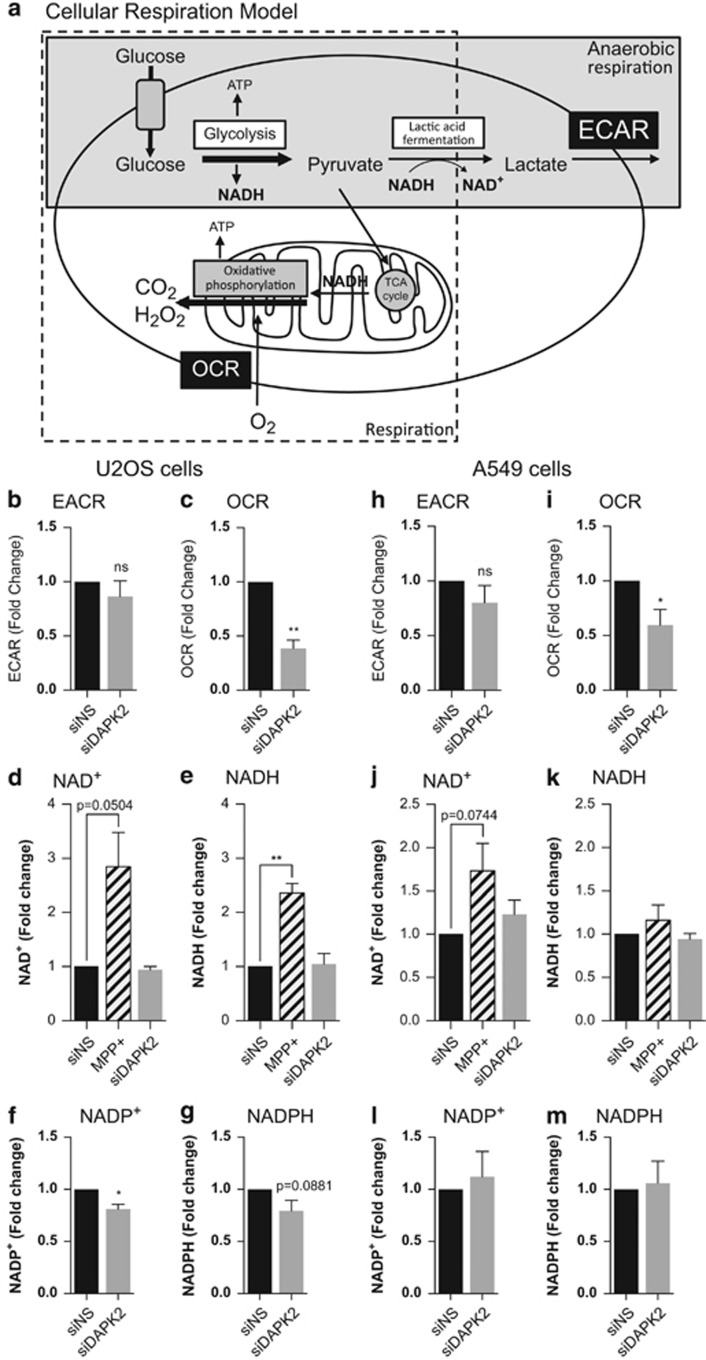
The absence of DAPK2 leads to reduced oxidative phosphorylation in U2OS and A549 cells. (**a**) Simplified cartoon depicting cellular metabolism pathways with glycolysis as the first step of glucose breakdown, and oxidative phosphorylation and anaerobic respiration as subsequent steps. To quantify cellular metabolic processes, U2OS (**b**–**g**) and A549 (**h**–**m**) cells were transfected with either siNS or DAPK2 siRNA. Forty-eight hours after transfection cells were analysed using a Seahorse Analyser. ECAR, an indirect measurement of lactic acid production, is depicted as fold change of mpH/min and normalised to siNS control in U2OS (**b**) and A549 cells (**h**). OCR, which can be used to determine mitochondrial respiration, is shown as fold change of pmol/min and normalised to siNS control in U2OS (**c**) and A549 cells (**i**). Forty-eight hours after siRNA transfection, NAD^+^, NADH, NADP^+^ and NADPH levels were analysed using colorimetric assays in U2OS (**d**–**g**) and A549 cells (**j**–**m**). Treatment with MPP+ (1 mM, 24 h) served as a positive control. Data represent mean±S.E.M. of three independent experiments, statistical analyses were done using Student's *t*-test (paired, one tailed) (*P<0.05, **P<0.01)

Eukaryotic cells use two key metabolic pathways for ATP generation (Figure 5a). Both pathways start with glycolysis as the first step of glucose metabolism, converting one glucose molecule into two molecules of pyruvate with gain of two ATP molecules. Aerobic respiration involves the transport of pyruvate into the mitochondria and mitochondrial respiration downstream of glycolysis through the tricarboxylic acid (TCA)/Krebs cycle, which yields another thirty four ATP molecules/molecule of glucose.^26^ Anaerobic respiration includes glycolysis and the fermentation of pyruvate to lactate, a metabolic pathway that bypasses mitochondrial respiration, and is predominantly upregulated in cancer cells (Warburg effect), which cancer cells use to produce most of their energy.^18, 27^

Changes in aerobic and anaerobic respiration were analysed by measuring the oxygen consumption rate (OCR), an accurate indicator of mitochondrial respiration, and the extracellular acidification rate (ECAR), an indirect measurement of lactic acid production, with a Seahorse XF96 analyser (Seahorse Bioscience, North Billerica, MA, USA; Figure 5). OCR and EACR were determined under basal conditions in siDAPK2- or siNS-transfected cells. The EACR in siDAPK2-tranfected cells, measured in mpH/min, was normalised to that in siNS-transfected control cells. No statistically significant changes in EACRs were detected between siNS- and siDAPK2-transfected U2OS (Figure 5b), or A549 (Figure 5h) cells, suggesting that silencing DAPK2 did not skew metabolism in these cells towards anaerobic respiration. OCR was measured in pMoles/min and normalised as described above for EACR measurements. In contrast to what was seen for EACR, there was a significant decrease in OCRs by mitochondria on DAPK2 knockdown in both U2OS (Figure 5c) and A549 (Figure 5i) cells, suggesting that DAPK2 modulated mitochondrial respiration but not anaerobic respiration.

To investigate further the consequences of DAPK2 depletion on cellular metabolism, levels of the coenzymes nicotinamide adenine dinucleotide (NAD^+^) and nicotinamide adenine dinucleotide phosphate (NADP^+^), as well as their corresponding reduced forms (NADH and NADPH, respectively), were analysed. During glycolysis and the TCA cycle, each glucose molecule leads to the reduction of six NAD^+^ to NADH, which are then oxidised during oxidative respiration to produce ATP.^26^ In contrast to NADH, NADPH is not involved in metabolic processes but it has an important role in the regeneration of oxidised glutathione (GSH), a key antioxidant in cells.^28^ Colorimetric assays were used to determine the levels of NAD^+^ and NADH, as well as NADP^+^ and NADPH. MPP^+^ was used to inhibit oxidative phosphorylation and increase the levels of NAD^+^ and NADH. As shown for U2OS (Figures 5d and e) and A549 cells (Figures 5j and k), the control MPP^+^ treatment increased the levels of NAD^+^ and NADH (*albeit* in A549 cells the increase in NADH was small and not statistically significant). However, NAD^+^/NADH levels remained unaffected by DAPK2 knockdown. Interestingly, the levels of NADP^+^ and NADPH in U2OS cells (Figures 5f and g) appeared slightly decreased on silencing DAPK2, whereas there was no detectable change in A549 cells (Figures 5l and m). The data thus suggested that RNAi-mediated ablation of DAPK2 affected oxidative phosphorylation without substantially impacting on NAD^+^/NADH or NADP^+^/NADPH metabolism.

### Depletion of DAPK2 leads to decreased GSH levels and induction of NRF2

**Figure 6:**
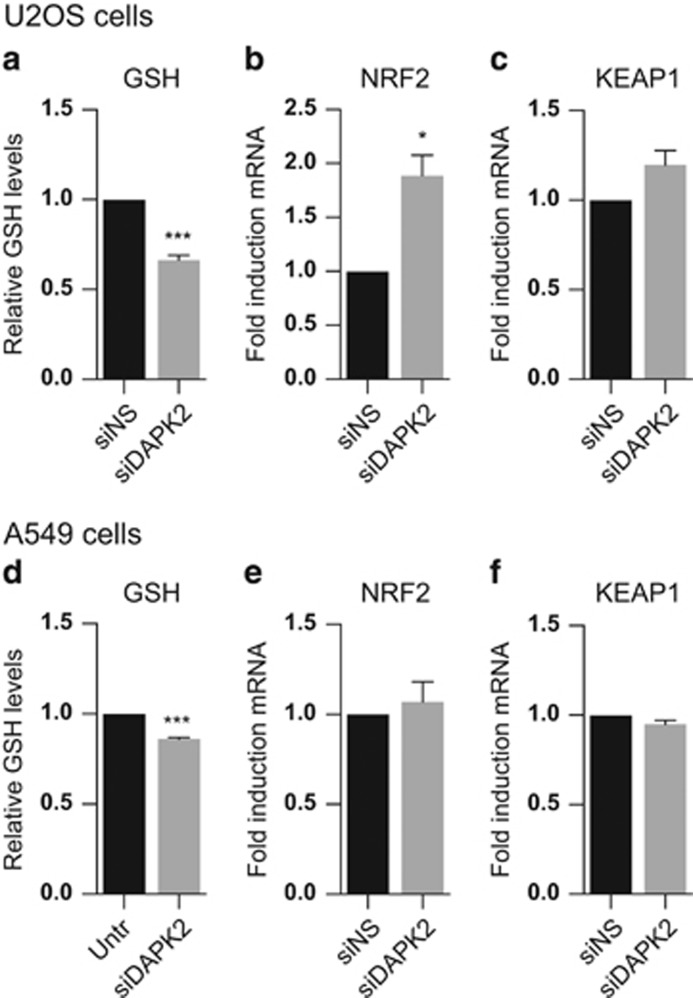
Ablation of DAPK2 leads to decreased GSH levels, and in U2OS cells to the induction of NRF2 mRNA. U2OS (**a**–**c**) and A549 (**d**–**f**) cells were transfected with either siNS or siDAPK2, and the levels of GSH were analysed using a colorimetric assay 24 h later (**a** and **d**). NRF2 (**b** and **e**) and KEAP1 (**c** and **f**) mRNA levels were assessed by qPCR. Data represent mean±S.E.M. of at least three independent experiments and the statistical analyses were done using Student's *t*-test (paired, one tailed) (*P<0.05, ***P<0.005)

Having established that genetic ablation of DAPK2 led to changes in cellular oxidative stress, mitochondrial respiration, activation of stress kinases and upregulation of SODs in two distinct cancer cell types, we next asked what effect downregulation of DAPK2 had on the levels of GSH, which functions as an electron donor and is involved in the reduction of cellular ROS.^29, 30^ In fact, the generation of oxidised GSH (GSSG) is tightly linked to mitochondrial oxidative phosphorylation, whereby NADPH reduces GSSG to GSH. To assess total GSH level, cells were transfected as described earlier and a subtle, but statistically significant, downregulation of GSH levels on DAPK2 knockdown was detected, which was more prominent in U2OS (Figure 6a) than in A549 cells (Figure 6e).

The question was whether such small reductions in GSH levels were sufficient to induce nuclear factor (erythroid-derived 2)-like 2 (NFE2L2/NRF2), which is responsible for the transcription of a multitude of antioxidants that protect cells from oxidative stress. NRF2 binds to the antioxidant response element, leading to transcriptional activation of GSH synthesising enzymes and other antioxidant enzymes, such as SOD1 and SOD2, which were upregulated on DAPK2 silencing (Figures 1b and f). After U2OS and A549 cells were transfected with siDAPK2 or siNS, RNA and proteins were extracted and the levels of NRF2 and its ubiquitin ligase kelch-like ECH-associated protein 1 (KEAP1) analysed. Interestingly, NRF2 mRNA levels were significantly elevated in U2OS cells (Figure 6b), but not in A549 cells (Figure 6e). KEAP1 was not induced in either U2OS (Figure 6c) or A549 cells (Figure 6f).

### The mitochondrial-associated function of DAPK2 is likely to be kinase mediated

The data thus far suggested that the increase in intracellular ROS caused by downregulating DAPK2 was due to an impairment of mitochondrial functions. To understand how DAPK2 preserved mitochondrial integrity, the relevance of DAPK2's kinase domain was investigated. For that purpose, tetracycline (Tet)-inducible U2OS cell lines (U2OS-TetR) containing HA-tagged DAPK2 constructs that were either wild-type (HA-DAPK2.wt) or kinase-dead (HA-DAPK2.K42A)^6^ were used. The caveat with this approach is that it was impossible to express the constructs at endogenous levels and overexpression of DAPK2 is sufficient to induce apoptotic features.^6^ This approach could not thus be used experimentally to rescue the phenotype observed after RNAi targeting DAPK2. It did, however, enable analysis of the role of the kinase domain on the regulation of intracellular oxidative stress.

**Figure 7:**
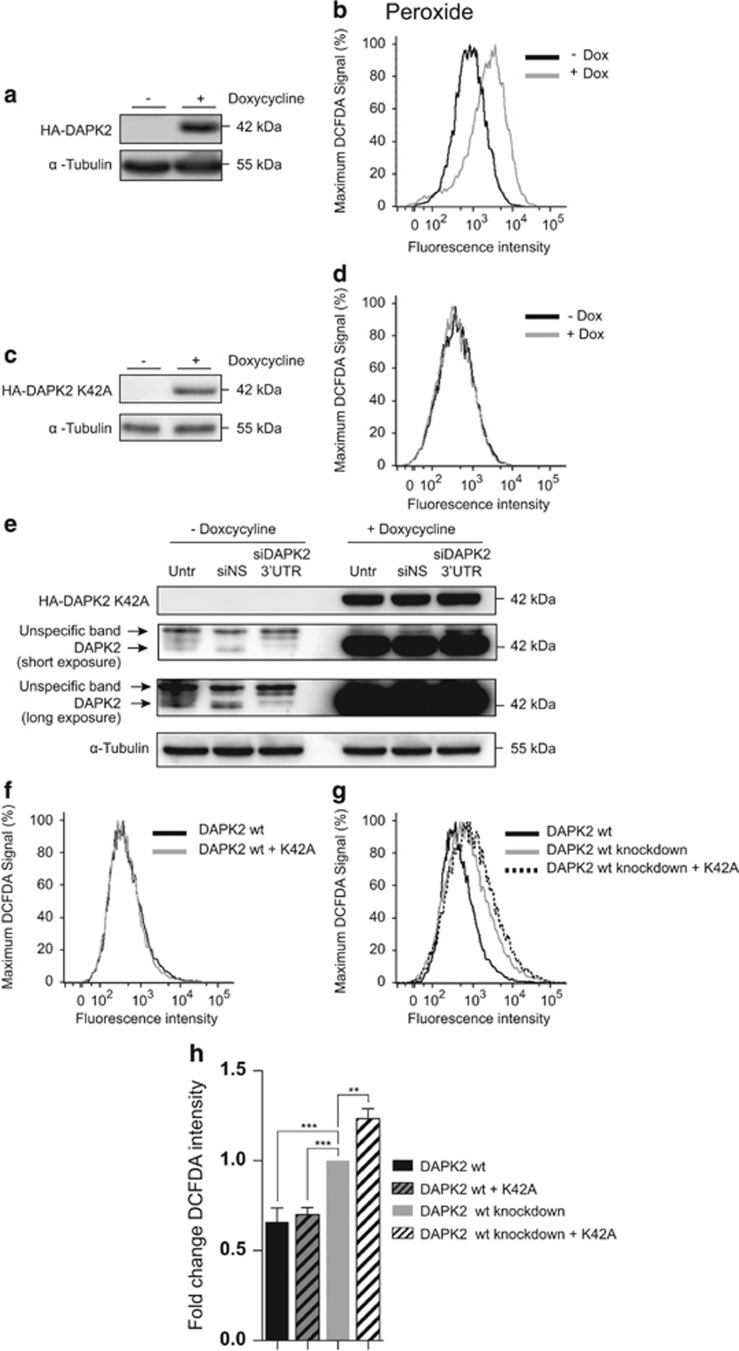
Effect of wild-type (wt) and kinase-dead (K42A) DAPK2 overexpression on oxidative stress. The expression of HA-tagged wild-type (wt) or HA-tagged kinase-dead (K42A) DAPK2 in U2OS-TetR cells was induced using doxycycline (10 ng/ml). HA-DAPK2 (**a**) and HA-DAPK2.K42A (**c**) expression was verified by SDS-PAGE/qWB. The impact of wild-type (**b**) or kinase-dead (**d** and **f**) DAPK2 overexpression on oxidative stress was analysed as described earlier using the DCFDA probe. To eliminate endogenous DAPK2 and study the effect of the kinase-dead DAPK2 mutant individually, U2OS-TetR cells containing the HA-DAPK2.K42A construct were transfected with either siNS or siDAPK2–3′UTR and concomitantly stimulated with doxycycline (10 ng/ml). Targeted knockdown of endogenous DAPK2 and overexpression of HA-DAPK2.K42A were then assessed by SDS-PAGE/qWB (**e**). The effect of the kinase-dead DAPK2 mutant overexpression, in the presence (**f,** grey line) and absence (**g,** dotted black line) of endogenous DAPK2, on oxidative stress was quantified by flow cytometry with the DCFDA probe (**f** and **g**). Data were plotted as fold change of DCFDA fluorescence of U2OS-TetR cell transfected with siDAPK2–3′UTR without doxycycline treatment (**h**). Statistical analysis was done using one-way ANOVA test (**P<0.01, ***P<0.005)

Transgene expression was induced in HA-DAPK2.wt (Figure 7a) and HA-DAPK2.K42A cells (Figure 7c) using doxycycline (Dox) and ROS levels were measured using DCFDA. As expected from previously published work, overexpression of wild-type DAPK2, rather than leading to reduced oxidative stress, led to an increase in general ROS production (Figure 7b), but importantly this was not observed if cells overexpressed a kinase-dead DAPK2 mutant (Figure 7d).

The overexpressed kinase-dead mutant (HA-DAPK2.K42A) did not behave as a dominant-negative and did not mimic the phenotype caused by DAPK2 silencing (Figure 7d). This might be due to the cells still expressing endogenous wild-type DAPK2. To eliminate the endogenous wild-type DAPK2 in the inducible cell model, 3′UTR-specific siRNAs were used. These molecules exclusively target endogenous DAPK2 (Figures 7e–h).^9^ Cells were transfected with either siNS or 3′UTR-specific DAPK2 siRNA (henceforth siDAPK2–3′UTR) and to induce HA-DAPK2.K42A expression cells were stimulated with Dox as they were being transfected. As shown in Figure 7e, siDAPK2–3′UTR specifically targeted endogenous DAPK2 and did not impact on the expression of kinase-dead DAPK2, which was detected using an anti-HA antibody.

Having established a cell system where it was possible to efficiently downregulate endogenous DAPK2 and concomitantly overexpress a kinase-dead DAPK2 mutant enabled studying the effect of kinase-dead DAPK2 on the generation of general ROS. Consistent with our previous results (Figure 1c), depletion of wild-type DAPK2 without (Figure 7g, grey line) or with overexpression of kinase-dead DAPK2 (Figure 7g, dotted line), using siDAPK2–3′UTR, led in both cases to a significant increase in ROS (Figures 7g and h). The overexpression of kinase-dead DAPK2, importantly, actually led to a significantly higher level of oxidative stress in U2OS cells than the wild-type endogenous protein (Figure 7h). In control cells transfected with siNS, the level of oxidative stress was the same regardless of the presence or absence of HA-DAPK2.K42A (Figure 7f). Collectively, our data suggested that DAPK2 kinase activity was important for preserving mitochondria's integrity and protecting cells from oxidative stress.

## Discussion

Although DAPK1–3 are structurally different, both DAPK2 and DAPK3 evolved from DAPK1,^31^ which regulates mitochondrial integrity^2^ and metabolic processes.^3^ Given the common ancestry and sequence homology within DAPKs' kinase domains, common mechanisms of action and functional redundancy have been postulated.^32^ Indeed, all three have been associated with apoptotic processes and thought to be potential tumour suppressors.^18^

Here we show that depletion of DAPK2 results in ROS generation (Figures 1c, d, g and h), induction of SODs (Figures 1b and f) and phosphorylation/activation of MAPKs (Figures 1a and e). Mitochondrial SOD2 was induced more than cytosolic SOD1 (Figures 1b and f), suggesting that in the absence of DAPK2, increased levels of ROS were produced by mitochondria.^13^ Additional experiments support this hypothesis (Figures 2a–c and e–g) and indicate that the ER is an unlikely additional ROS source (Figures 2d and h). Silencing DAPK2 leads to increased spontaneous mitochondrial depolarisation (Figures 3a–l and Figure 4), and in A549 cells to the activation of DAPK1 (Figure 3m), thought to be a Δ*ψ*m sensor involved in cytoskeletal rearrangements responsible for mitochondrial transport mechanisms.^2^ DAPK1 and DAPK2 have also been reported to heterodimerise.^32^ It is thus reasonable to assume that they may operate similarly with regard to mitochondrial maintenance, sensing the mitochondrial membrane potential and leading to downstream processes of mitophagy and mitochondrial degradation, which result in faulty mitochondria. Interestingly, neither overexpression of DAPK1 nor DAPK2 has been shown to induce mitochondrial depolarisation.^6^ Immunofluorescence analyses using MitoTracker Red did not suggest any obvious mitochondrial morphological changes in cells devoid of DAPK2 (data not shown).

DAPK2 knockdown leads to significantly reduced oxidative phosphorylation without affecting lactic acid fermentation (Figures 5b, c, h and i). This suggests a fundamental role for DAPK2 in the regulation of mitochondrial metabolism and/or maintenance. This, in turn, raises the question of how these cells maintain their energy supply, without increased lactic acid fermentation, DAPK2-depleted cells are unlikely to be compensating the decrease in oxidative phosphorylation via anaerobic respiration (Figure 5a). Consistent with this, no significant changes in NAD^+^/NADH and/or NADP^+^/NADPH on DAPK2 silencing are seen (Figures 5d–g and j–m). All experiments were performed 48 h post RNAi. Thus, eventual cellular compensatory mechanisms, such as increased glycolytic rate or lactic acid production, may not yet be detectable. For example, it has been shown that inhibition of the mitochondrial electron transport by low doses of ethidium bromide for 4 days causes NADH accumulation, halts the TCA cycle and drives cells towards anaerobic glucose metabolism.^33^

The ROS increase and mitochondrial depolarisation were accompanied by a small, statistically significant, GSH decrease (Figures 6a and d), and in U2OS cells by the induction of NRF2 (Figure 6b). There was no induction of NRF2 in A549 cells (Figure 6e) and the levels of KEAP1, a NRF2 regulator,^34^ remained unchanged in both cell lines (Figures 6c and f). SOD2 protein expression levels, unlike what was observed at the mRNA level, did not vary with the downregulation of DAPK2 expression. In fact, the expression of GCLC, GPX1, GSTpi and GSS, enzymes involved in GSH metabolism, did not change significantly either on RNAi against DAPK2 (data not shown). These data thus suggest that it is their enzymatic activity that matters, which is corroborated by the GSH quantification shown in Figures 6a and e, used as an indirect read-out for these enzymes.

Overexpression of HA-tagged wild-type DAPK2 *per se* increases the levels of intracellular ROS in U2OS cells (with endogenous DAPK2), whereas kinase-dead DAPK2 does not (Figures 7a–d). In contrast, as previously shown,^6^ overexpression of wild-type DAPK2 does not induce mitochondrial ROS (data not shown). Interestingly, overexpression of the kinase-dead mutant, in the absence of endogenous DAPK2, leads to increased oxidative stress and this is greater than that seen with the wild-type protein (Figures 7e–h). Collectively, these data indicate that DAPK2 kinase activity is important for preserving mitochondrial integrity and protecting cells from oxidative stress.

DAPK2 is emerging as a tumour suppressor in several types of cancer cells, especially in acute promyelocytic leukaemia, which responds to treatment with all-trans retinoic acid (ATRA).^12^ This is interesting as ATRA leads to increased DAPK2 expression. Given the role of ROS in the aetiology of AML,^35^ downregulation and/or loss of DAPK2 likely benefits cancer cells by leading to deregulated mitochondria, increased cellular ROS and, thus, genomic instability. Indeed, somatic mitochondrial mutations and accumulated mitochondrial damage have been linked to AML development.^36^ Despite its relatively small size and straightforward structural features, DAPK2 appears to be a double-edge sword, depending on cellular context and interaction partners. Indeed, recently, we have shown that downregulation of endogenous DAPK2 activates NF-*κ*B and sensitises multiple cancer cell types to TRAIL-induced apoptosis but not to other cytotoxic stimuli.^9^ NF-*κ*B, another multi-faceted protein, can also be activated by oxidative stress but TRAIL-induced apoptosis appears to be independent of oxidative stress since it can be blocked by caspase inhibitors (Supplementary Figure S2), but these inhibitors do not impair the production of ROS (Supplementary Figure S3) or mitochondrial depolarisation (Supplementary Figure S4) seen after DAPK2 depletion. Experiments using U2OS cells that express LC3–GFP (an autophagy readout) suggest that depletion of DAPK2 also affects autophagy, as in these cells nutrient starvation leads to an increase in the expression of LC3I (data not shown). It is still unclear if this directly impairs mitochondrial recycling, leading to the accumulation of ‘faulty' mitochondria, and if it contributes for the oxidative stress measured here. This is entirely consistent with a novel report by Kimchi and co-workers,^37^ where it is shown that DAPK2 is a novel mTORC1 kinase and therefore a novel regulator of autophagic processes. How this relates to the regulation of NF-*κ*B^9^ remains under scrutiny.

We have thus identified a novel role for DAPK2 in the regulation of mitochondrial integrity, as its absence leads to mitochondrial depolarisation and increased oxidative stress. This may be a mechanism by which, depending on the intracellular context, DAPK2 can act as a tumour suppressor gene. Importantly, as the effect of DAPK2 silencing on mitochondrial respiration is conserved between mesenchymal U2OS cells and epithelial A549 cells, it is likely that our findings can be further extended to additional cell lineages and malignancies.

## Materials and Methods

### Cell culture

U2OS and A549 cells were grown in Dulbecco's Modified Eagle's medium (DMEM) supplemented with 10% (v/v) foetal calf serum (FCS) (FirstLink, Wolverhampton, UK), 2 mM L-glutamine, 50 U/ml penicillin and 50 *μ*g/ml streptomycin (henceforth referred to as ‘complete DMEM'), in a humidified atmosphere of 10% CO_2_ at 37 °C. The U2OS-TetR cell line was provided by Professor SA Johnsen^38^ and cultured in full DMEM media supplemented with 5 *μ*g/ml blasticidine S hydrochloride. All chemicals were from Sigma-Aldrich (St. Louis, MO, USA), unless otherwise specified.

### Plasmids

N-terminal HA-tagged wild-type and kinase-dead (K42A) DAPK2 pcDNA3 plasmids were kindly provided by Kimchi and co-workers.^6^ Both constructs were sub-cloned using Bam*HI* and Xh*oI* restriction sites into a pcDNA4/TO vector, which is part of the Tet-inducible mammalian expression system, T-REx System (Life Technologies, Paisley, UK).

### Generation of stable inducible cell lines

HA-tagged DAPK2 wild-type and kinase-dead (K42A) constructs, cloned into the pcDNA4/TO vector, were linearised using the restriction enzyme *PvuI* (New England Biolabs, Hitchin, UK) and transfected into U2OS-TetR cells using Lipofectamine 2000 (Life Technologies), as instructed by the manufacturer. Forty-eight hours later, cells were re-plated and incubated with complete DMEM supplemented with 5 *μ*g/ml blasticidine S hydrochloride and 500 *μ*g/ml Zeocin (Life Technologies). Individual clones were isolated using cloning cylinders and tested for induction of the transgene and background expression by SDS-PAGE/qWB. For each construct, at least five inducible clones were generated and tested.

### Antibodies

The anti-DAPK2 antibody was purchased from Epitomics (Burlingame, CA, USA); anti-phospho-DAPK1 (Ser308) and DAPK1 from Abcam (Cambridge, UK); antibodies against phospho-ERK1/2 (Thr202/Tyr204), phospho-p38 MAPK (Thr180/Tyr182), phospho-JNK (Thr183/Tyr185) and CHOP from Cell Signaling Technology (Cambridge, UK); antibodies against HA, *β*-actin and *α*-tubulin from Sigma-Aldrich; anti- HSP90 (HSP86) from NeoMarkers (Fremont, CA, USA); antibodies against ERK1/2 and Lamin B were from Santa Cruz Biotechnology (Santa Cruz, CA, USA). Anti-mouse and anti-rabbit secondary antibodies were from DAKO (Glostrup, Denmark) and the anti-goat antibody was from Sigma-Aldrich.

### RNA interference

RNAi was performed using Lipofectamine RNAiMax (Life Technologies), essentially as described in ref. 39. For forward siRNA transfection cells, plated at a density of 2.5 × 10^5^ in six-well plates, were left untransfected, or were transfected with either 20 nM AllStars non-targeting control siRNA (siNS) (Qiagen, Hilden, Germany), 20 nM of a siDAPK2 pool (siRNA oligonucleotides 3 and 4 from Dharmacon, Lafayette, CO, USA) or with either of two different 3′UTR-specific siRNAs (from Dharmacon or Qiagen). Cells were then treated and analysed as described in the figures using methods described below. Reverse siRNA transfections were carried out in 10 cm dishes using 5 × 10^5^ cells in suspension (8 ml). Cells were left untransfected or were mixed with transfection reagent and either 20 nM siNS or siDAPK2. All siRNA oligonucleotide sequences are listed on Supplementary Table SI.

### Protein expression analysis

Proteins were extracted using radioimmunoprecipitation assay buffer (50 mM Tris-HCl pH 7.4; 0.5% (v/v) NP-40, 150 mM NaCl, 1 mM EDTA, 1 mM Na_3_VO_4_ and cOmplete and Mini, EDTA-free protease inhibitor cocktail, the latter used as instructed by the manufacturer (Roche, Mannheim, Germany)). Concentrations were determined using a Bradford assay according to the manufacturer's instructions (BioRad, Hercules, CA, USA). They were then analysed by SDS-PAGE/ qWB. Membranes were blocked using 5% (w/v) non-fat milk/TBS-Tween 20, which was the buffer also used to dilute secondary antibodies. Primary antibodies were diluted in 5% (w/v) BSA/ TBS-Tween 20. WBs were analysed using the quantitative luminescence system FUSION Solo (PEQLAB, Sarisbury Green, UK). Images were analysed using Image Studio Lite software (LI-COR Biosciences, Lincoln, NE, USA) (www.licor.com/islite). The WB images have been cropped for clarity purposes: that has in no way altered the essence of the data obtained in three independent experiments.

### Detection of oxidative stress by flow cytometry

Cells were cultured in six-well plates and transfected with siRNA oligonucleotides as described before, or treated with 0.5 mM H_2_O_2_ for 24 h, which was used as a positive control for ROS production. General ROS were detected using CM-H_2_DCFDA (Molecular Probes, Life Technologies, Paisley, UK; referred throughout the text as DCFDA). For that purpose, cells were incubated with 10 *μ*M DCFDA dissolved in pre-warmed PBS for 30 min at 37 °C. Cells were then trypsinised, resuspended in complete DMEM, washed twice with PBS and analysed by flow cytometry. Cellular superoxide anions were detected using DHE (Molecular Probes) 48 h after transfection. Essentially, cells were trypsinised, resuspended in complete DMEM, washed twice with PBS, incubated in 10 *μ*M DHE dissolved in pre-warmed PBS for 15 min and analysed by flow cytometry. Mitochondrial superoxide anions were measured using the MitoSOX Red mitochondrial superoxide indicator. Briefly, cells were transfected as described earlier, or they were treated for 24 h with 1 mM MPP^+^, or with 0.5 mM H_2_O_2_, the latter two used as positive controls. Forty-eight hours later, cells were washed with PBS and incubated for 10 min at 37 °C in the dark with 5 *μ*M MitoSOX Red reagent diluted in pre-warmed PBS. Samples were subsequently washed twice with PBS before being analysed. Depolarised mitochondria were quantified using the MitoProbe 5′,6,6′-tetrachloro-1,1′,3,3′-tetraethylbenzimidazolylcarbocyanine iodide (JC-1) Assay Kit and Tetramethylrhodamine, ethyl Ester, perchlorate (TMRE; Molecular Probes). For JC-1 staining, 48 h after transfection, cells were trypsinised, resuspended in complete DMEM, washed twice with PBS and incubated with 2 *μ*M JC-1 dissolved in pre-warmed PBS at 37 °C, 10% CO_2_ for 15 min and analysed by flow cytometry. TMRE staining was performed as follows: 48 h after transfection cells were incubated with 100 nM TMRE dissolved in pre-warmed complete DMEM at 37 °C, 10% CO_2_ for 10 min and analysed by flow cytometry. To induce complete mitochondrial depolarisation, the protonophore CCCP (50 mM) was used for 5 min prior to the incubation of cells with JC-1 and TMRE.

All flow cytometry analyses were performed using a FACS Canto (Becton Dickinson, Franklin Lakes, NJ, USA) and data mining was done using FlowJo (Tree Star, Inc., Ashland, OR, USA). Fluorescence geometric means were used for the analyses and samples were normalised to siNS-transfected control cells, essentially as described earlier.^39^

### Induction of ER stress

ER stress was induced using the glycosylation inhibitor tunicamycin (0.5 *μ*g/ml, 24 h) and monitored by SDS-PAGE/qWB using CHOP expression as a read-out.

### Real-time quantitative PCR

Gene expression analysis was done by quantitative two-step reverse transcription PCR. Reverse transcription was performed using total RNA and a High Capacity cDNA Reverse Transcription kit (Life Technologies), using random hexamers. Quantitative PCR (qPCR) was done using the Fast SYBR Green Master Mix (Life Technologies) with specific primer pairs (Supplementary Table SII). For each target mRNA analysed, 2.5 *μ*l of Fast SYBR Green Master Mix, 0.5 *μ*M of each primer pair and 2 *μ*l of cDNA in deionised water (5 ng/*μ*l) were mixed in 384-well plates in duplicates using Matrix Equalizer Electronic Multichannel Pipetters (Thermo Fisher Scientific, Loughborough, UK). qPCR was carried out on an ABI PRISM 7900HT (Applied Biosystems, Foster City, CA, USA) using the following settings: initial activation of 20′′ at 95 °C, 40 cycles; denaturation for 1′ at 95 °C; annealing/extension for 20′′ at 60 °C; final melting curve was carried out for 15′′ at 95 °C and then for 15′′ at 60 °C. Quantification of target messages was performed using qbasePLUS software (Biogazelle, Ghent, Belgium). HPRT and GAPDH were the reference genes used for normalisation.

### Metabolic analyses

Metabolic measurements were done in real-time, non-invasively, using a Seahorse XF96 analyser, which measured the OCR and the ECAR under basal conditions in siDAPK2- or siNS-transfected cells. A cell titration assay was used to determine a suitable cell plating density for both A549 and U2OS cells. For the experiment proper, 0.5 × 10^6^ cells were plated per 10 cm dish and reverse transfected on day 1 as previously described. Forty-eight hours post transfection, cells were re-plated into the XF96 microplates (Seahorse Bioscience; 4 × 10^4^ cells per 100 *μ*l per well) and incubated overnight at 37 °C. The XF calibration solution (Seahorse Bioscience) was added into the XF sensor cartridge (Seahorse Bioscience) and was also incubated at 37 °C overnight but without CO_2_. The next day, prior to the assay, complete DMEM was replaced with an XF Assay Medium Modified DMEM (Seahorse Bioscience) (1 g/ml glucose, pH 7.4) and cells were incubated at 37 °C for 1 h without CO_2_. Analyses were performed according to the manufacturer's instructions using eight measurements that the instrument recorded for OCR (nmoles/min) and ECAR (mpH/min) pertaining to each well. Results were analysed using the provided XFe Wave software (Seahorse Bioscience).

### GSH colorimetric assay

Cells (2.5 × 10^5^ per well in 6-well plates) were transfected with siRNA oligonucleotides as described earlier. Cell lysates were collected and sonicated in 50 mM PBS per 1 mM EDTA. Protein concentrations were determined using a Bradford assay, according to the manufacturer's instructions, which enabled subsequent normalisation of each sample so that the final protein concentration was 1 *μ*g/*μ*l. GSH levels in each sample were quantified using the Glutathione Assay Kit from BioAssay Systems (Hayward, CA, USA), as per the manufacturer's protocol.

### NADH and NADPH assay

Reverse siRNA transfections were carried out in 10-cm dishes using 5 × 10^5^ cells in suspension. As positive control, cells were treated for 24 h with 1 mM of MPP^+^. Forty-eight hours after transfection, NADP/ NADPH levels for each sample were quantified using the NADP/NADPH assay kit (Abcam) and NAD/NADH levels using the NAD/NADH assay kit (BioVision, San Francisco, CA, USA), according to the two manufacturers' protocols.

### Statistical analysis

Mean±S.E.M. of at least three independent experiments were used throughout. ANOVA or *t*-test statistical analyses were carried out as indicated in each figure legend using GraphPad Prism (GraphPad Software Inc., San Diego, CA, USA).

## Abbreviations

ATRA: all-trans retinoic acid
CCCP: carbonyl cyanide 3-chlorophenylhydrazone
CHOP: CCAAT-enhancer-binding protein homologous protein
CM-H2DCFDA: chloromethyl 2′,7′-dichlorodihydrofluorescein diacetate
DAPK: death-associated protein kinase
DHE: dihydroethidium
DMEM: Dulbecco's Modified Eagle's medium
Dox: doxycycline
ECAR: extracellular acidification rate
ER: endoplasmic reticulum
GSH: glutathione
GSSG: oxidised GSH
KEAP1: kelch-like ECH-associated protein 1
MAPK: mitogen-activated protein kinase
MPP^+^: mitochondrial complex I inhibitor 1-methyl-4-phenylpyridinium
NAD^+^: nicotinamide adenine dinucleotide
NADP^+^: nicotinamide adenine dinucleotide phosphate
NFE2L2/NRF2: nuclear factor (erythroid-derived 2)-like 2
OCR: oxygen consumption rate
qWB: quantitative western blots
RNAi: RNA interference
ROS: reactive oxygen species
SDS-PAGE: sodium dodecyl sulphate-PAGE
siNS: non-targeting siRNA oligonucleotide
siRNA: short interfering RNA
SOD: superoxide dismutase
TCA: tricarboxylic acid
Tet: tetracycline
TMRE: tetramethylrhodamine ethyl ester
Δ*ψ*m: mitochondrial membrane potential
