# Supplementary material for: DAPK2 regulates oxidative stress in cancer cells by preserving mitochondrial function
Source: Cell Death Dis. 2015 Mar 5;6(3):e1671–. doi: 10.1038/cddis.2015.31 (PMC4385915; doi:10.1038/cddis.2015.31)
Supplement: Supplementary Information [file cddis201531x1.pdf]

Supplementary Figure 1. Schlegel 2014

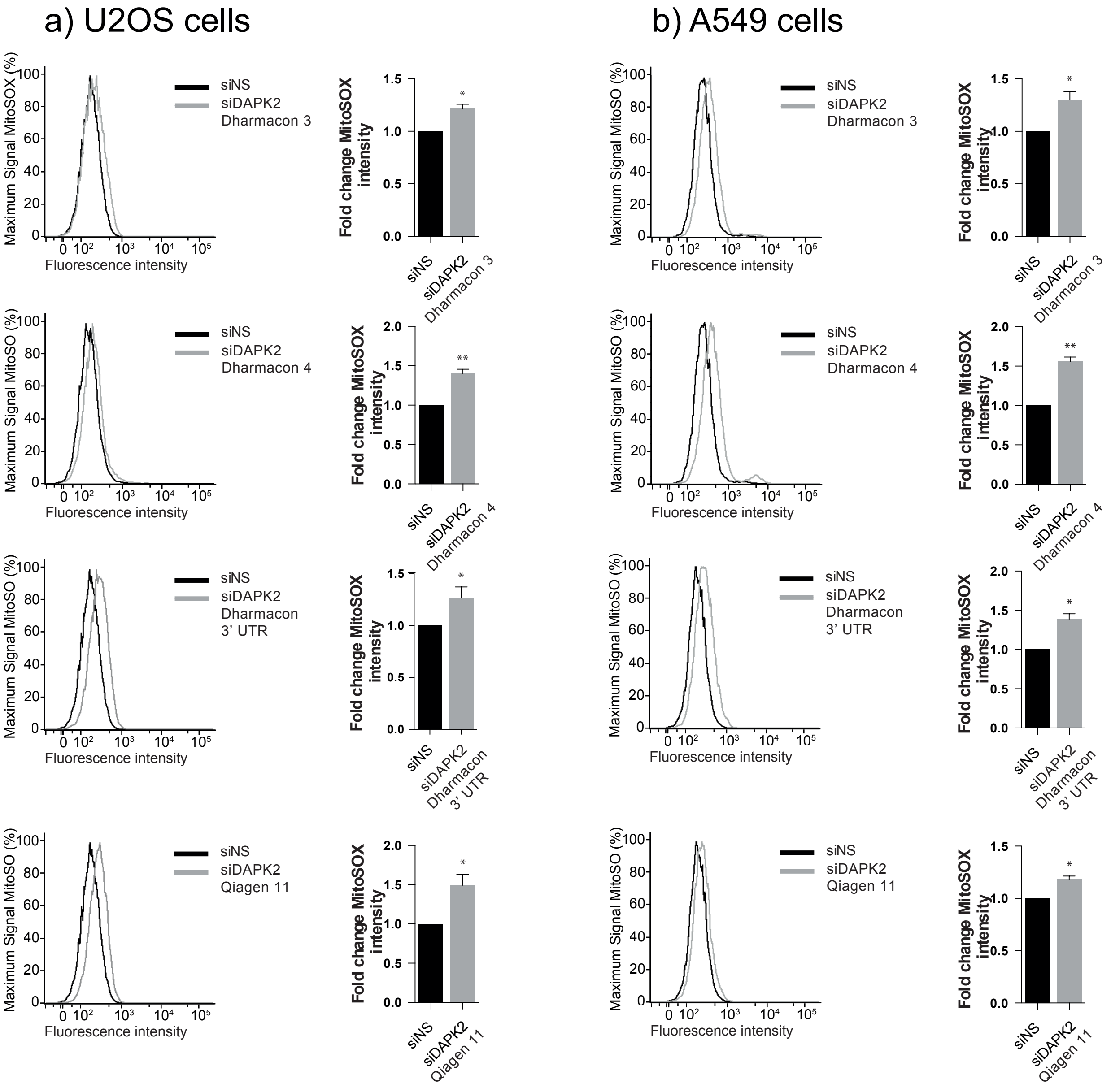

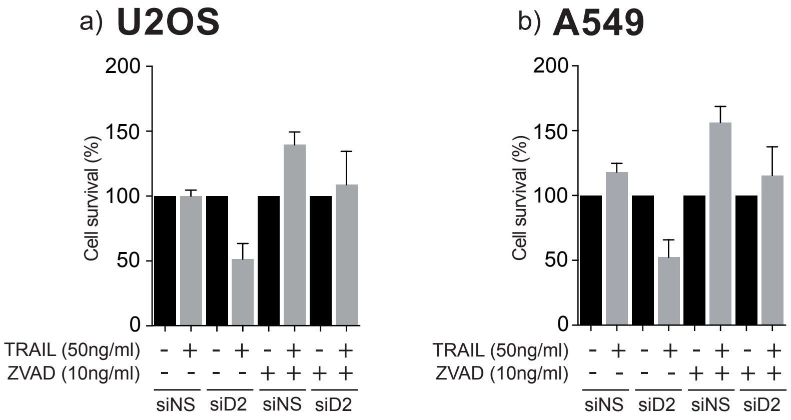
a) U2OS
b) A549
Cell survival (%)
200
150
100
50
0
TRAIL (50ng/ml) - + - + - + - +
ZVAD (10ng/ml) - - - - + + + +
siNS siD2 siNS siD2

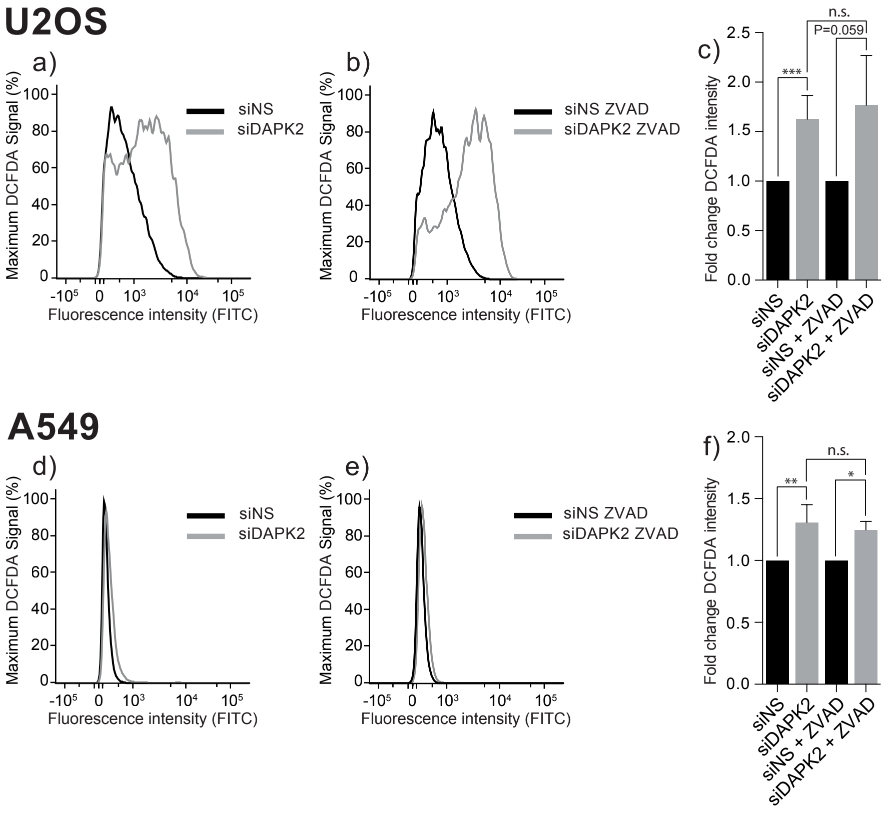
U2OS
a)
b)
c)
A549
d)
e)
f)
Maximum DCFDA Signal (%)
Fluorescence intensity (FITC)
siNS
siDAPK2
siNS ZVAD
siDAPK2 ZVAD
Fold change DCFDA intensity
siNS
siDAPK2
siNS + ZVAD
siDAPK2 + ZVAD
n.s.
P=0.059
***
**
*

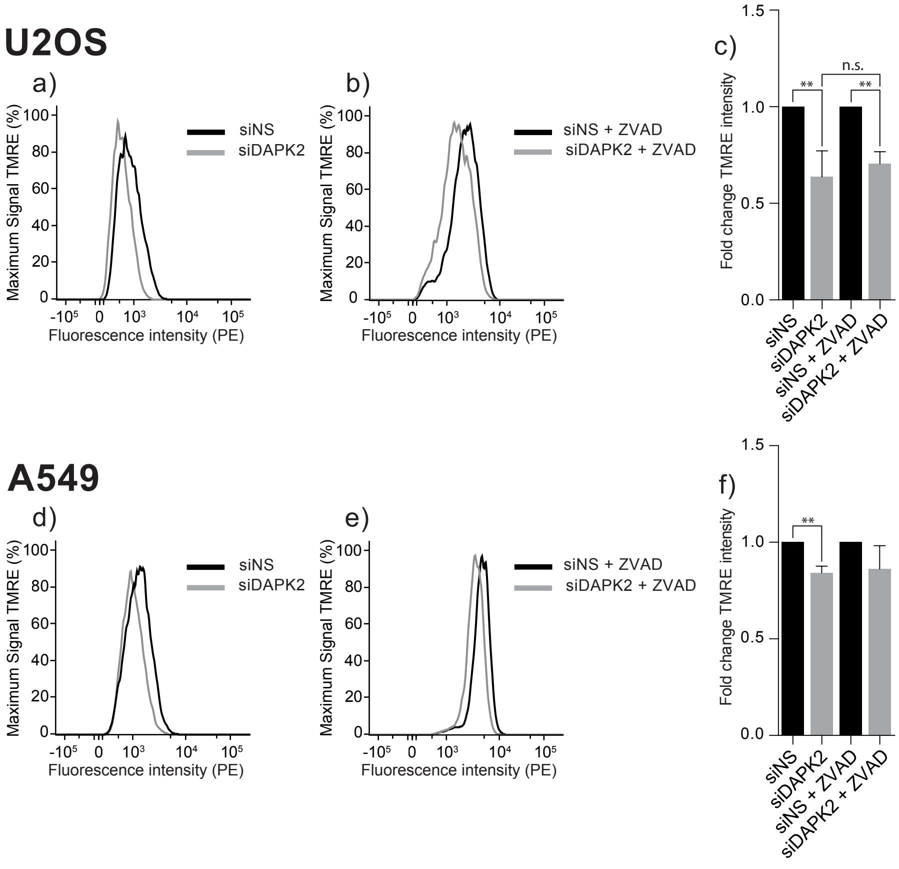
U2OS
a)
b)
c)
A549
d)
e)
f)
siNS
siDAPK2
siNS + ZVAD
siDAPK2 + ZVAD
Maximum Signal TMRE (%)
Fluorescence intensity (PE)
Fold change TMRE intensity
n.s.
**

Supplementary Figure Legends

Supplementary Figure S1. Effect of deconvoluted DAPK2 siRNA on mitochondrial $O_2^{\bullet-}$. Four different siRNAs targeting DAPK2 were used in this work and the effect of each individual siRNA oligonucleotide on the generation of mitochondrial $O_2^{\bullet-}$ each was tested by flow cytometry. U2OS **(a)** and A549 **(b)** cells were transfected with either siNS, or each individual siRNA oligonucleotide targeting DAPK2. Forty-eight h after transfection, the production of mitochondrial $O_2^{\bullet-}$ was assessed using the MitoSOX™ Red probe. Staining intensity was quantified using geometric means of at least three independent experiments and plotted as fold change in relation to the siNS-transfected control cells. Statistical analysis was done using Student's *t*-test (paired, two tailed) (* p< 0.05, ** p< 0.01).

Supplementary Figure S2. Sensitisation of resistant cancer cells to TRAIL after silencing DAPK2 is caspase-dependent. Four hours after U2OS **(a)** and A549 **(b)** cells were transfected with either siNS, or DAPK2 siRNA, they were incubated in ZVAD-containing media (10 ng/ml), which due to its half-life was added fresh to cells every day throughout the experiment. The next day, cells were re-plated in 96 well plates at a density of 2x104 cells in fresh ZVAD-containing media, or media devoid of ZVAD. The following day cells were treated with TRAIL (50 ng/ml) in the presence or absence of ZVAD for 24 h. Cells were then fixed using methanol and stained with crystal violet. Crystals were dissolved in 10% (v/v) acetic acid and quantified by measuring the absorbance at 595 nm. Values were normalised to the untreated samples. Data represent mean ± SEM of two independent experiments performed in triplicates.

Supplementary Figure S3. Oxidative stress in response to silencing DAPK2 is caspase independent. U2OS (**a-c**) and A549 (**d-f**) cells were transfected with either siNS, or DAPK2 siRNA. Four h after transfection, media was changed to ZVAD (10 ng/ml) containing media (**b, e**). Due to the short half-life of ZVAD, ZVAD-containing media was

renewed the next day. Forty-eight h after transfection, induction of oxidative stress was measured using a flow cytometer and the DCFDA probe. Staining intensity was quantified using geometric means of three independent experiments and plotted as fold change (**c**, **f**). Data represent mean ± SD of three independent experiments and the statistical analysis was done using Student's *t*-test (paired, one tailed) (* p< 0.05, ** p<0.01, *** p<0.005).

Supplementary Figure S4. siDAPK2-induced depolarisation of the mitochondria cannot be rescued by inhibiting the caspases using ZVAD. U2OS (**a-c**) and A549 (**d-f**) cells were transfected with either siNS, or DAPK2 siRNA as described in Appendix Fig. 3. Mitochondrial membrane depolarisation was assessed using the TMRE probe by measuring the fluorescence intensity in the red fluorescence channel (PE) of a flow cytometer. Staining intensity was quantified using geometric means of three independent experiments and plotted as fold change (**c**, **f**). Data represent mean ± SD of three independent experiments and the statistical analysis was done using Student's *t*-test (paired, one tailed) (* p< 0.05, ** p<0.01). Data for ZVAD treated A549 cells represents two independent experiments.

Supplementary Tables

TABLE SI. siRNA oligonucleotide sequences used throughout this study

Target gene/ Oligonucleotide name	Target sequence	Supplier/ Product number
Non targeting (All Stars)	Not disclosed by Qiagen	Qiagen-SI03650318
DAPK2_3	GGAAACGGCUCACAAUCCA	Dharmacon-004418-03
DAPK2_4	GGAAUUUGUUGCUCCAGAA	Dharmacon-004418-04
DAPK2_3'UTR	GAGUGUGGACUUAGGAAAA	Dharmacon-custom
DAPK2_Qiagen 11	CAGCAUUCCCAAAGCTCUU	Qiagen-SI04988298

TABLE SII. Oligonucleotide qPCR primer pair sequences used throughout this study

Oligonucleotide name	Oligonucleotide sequence (5' → 3')	Published source
DAPK2 fwd	TCCTGGATGGGGTGAACTAC	
DAPK2 rev	CAGCTTGATGTGTGGAATGG	
GAPDH fwd	AGCCACATCGCTCAGACAC	
GAPDH fwd	GCCCAATACGACCAAATCC	
HPRT fwd	TGACCTTGATTTATTTTGCATACC	
HPRT rev	CGAGCAAGACGTTCAGTCCT	
KEAP1 fwd	CAGATTGGCTGTGTGGAGTT	
KEAP1 rev	GCTGTTCGCAGTCGTACTTG	
NRF2 fwd	GAGAGCCCAGTCTTCATTGC	1
NRF2 rev	TTGGCTTCTGGACTTGGAAC	
p21 fwd	TGAGCCGCGACTGTGATG	2
p21 rev	GTCTCGGTGACAAAGTCGAAGTT	
p53 fwd	TAACAGTTCCTGCATGGGCGGC	3
p53 rev	AGGACAGGCACAAACACGCACC	
SOD1 fwd	AGGGCATCATCAATTTCGAG	
SOD1 rev	TGCCTCTCTTCATCCTTTGG	
SOD2 fwd	CGACCTGCCCTACGACTACG	
SOD2 rev	TGACCACCACCATTGAACTT	

[1]Lister A *et al.* (2011). *Molecular cancer* **10**: 37.
[2]Chen X *et al.* (2013). *BMC biology* **11**: 73.
[3]Chew YC *et al.* (2012). *The journal of biological chemistry* **287**: 16168-16178.
